# Experimental infection of Pacific oyster *Crassostrea gigas *spat by ostreid herpesvirus 1: demonstration of oyster spat susceptibility

**DOI:** 10.1186/1297-9716-42-27

**Published:** 2011-02-07

**Authors:** David Schikorski, Tristan Renault, Denis Saulnier, Nicole Faury, Pierrick Moreau, Jean-François Pépin

**Affiliations:** 1 Ifremer (Institut Français de Recherche pour l'Exploitation de la Mer), Laboratoire de Génétique et Pathologie (LGP), 17390 La Tremblade, France

## Abstract

In 2008 and 2009, acute mortalities occurred in France among Pacific cupped oyster, *Crassostrea gigas*, spat. Different hypothesis including the implication of environmental factors, toxic algae and/or pathogens have been explored. Diagnostic tests indicated that OsHV-1 including a particular genotype, termed OsHV-1 μVar, was detected in most of samples and especially in moribund oysters with the highlighting of virus particles looking like herpes viruses by TEM examination. In this study, an experimental protocol to reproduce OsHV-1 infection in laboratory conditions was developed. This protocol was based on the intramuscular injection of filtered (0.22 μm) tissue homogenates prepared from naturally OsHV-1 infected spat collected on French coasts during mortality outbreaks in 2008. Results of the experimental trials showed that mortalities were induced after injection. Moreover, filtered tissue homogenates induced mortalities whereas the same tissue homogenates exposed to an ultraviolet (UV) treatment did not induce any mortality suggesting that oyster spat mortalities require the presence of a UV sensitive agent. Furthermore, analysis of injected oyster spat revealed the detection of high amounts of OsHV-1 DNA by real-time quantitative PCR. Finally, TEM analysis demonstrated the presence of herpes virus particles. The developed protocol allowed to maintain sources of infective virus which can be useful for the development of further studies concerning the transmission and the development of OsHV-1 infection.

## Introduction

World mollusc aquaculture is characterised by a focus on a limited number of species including oysters being raised at an industrial level. The oyster industry has grown to be very important for many regions of the world contributing substantially to social and economic activity in the coastal zones. The yearly European oyster production is the 126 000 tonnes, France being the leading Member State (115 000 tonnes/year) [1]. The French oyster production is essentially based on the rearing of the Pacific cupped oyster, *Crassostrea gigas*. This species has been introduced in France from Canada and Japan in the early 70's after the extinction of the Portuguese oyster *C. angulata *related to irido-like virus infections [2].

However, since the late 80's abnormal mortality events have been reported in France among *C. gigas *oysters in the field and in hatcheries/nurseries [3-5]. These mortalities were usually sudden and severe (up to 100%), and affected essentially spat (oysters less than one year old) and juveniles (12 to 18 month old oysters). Mortality outbreaks took place currently during the summer period from mid May to July concomitantly with a rapid increase of seawater temperature (Garcia et al., unpublished data). These recurrent mortality events have been mainly associated to the detection of the ostreid herpesvirus 1 (OsHV-1), the sole member of the *Malacoherpesviridae *family [6-8].

The first description of a virus morphologically similar to herpes viruses was reported by Farley et al. [9] in the eastern oyster *Crassostrea virginica*. Herpes-like virus infections have then been noticed worldwide in several other mollusc species in association with massive mortality episodes, such as in oysters [10-17], in clams [11,18], in scallops [19] and in abalone [20-22]. Data available in literature [4] and those collected during an epidemiological survey conducted by the French National Network for Surveillance and Monitoring of Mollusc Health (Repamo) between 1997 and 2006 suggested a causal link between spat mortality and OsHV-1 detection in France (Garcia et al., unpublished data). Results of diagnostic tests indicated that OsHV-1 was detected in most of samples collected during mortality outbreaks and especially in moribund oysters (Garcia et al., unpublished data). To date, the infectivity of OsHV-1 was univocally demonstrated towards early stages of *C. gigas *through experimental trials. Experimental transmission assays have demonstrated that healthy larvae could be infected by contact with filtered (0.22 μm) tissue homogenates prepared from infected larvae [10,11,23,24]. So far, assays to reproduce the virus disease in experimental conditions on oyster spat have been inconclusive [25].

Massive mortality outbreaks in *C. gigas *oysters were reported in France, Ireland and the Channel Islands in 2008 and 2009 resulting in a shortage in supplies of the shellfish over the next years. Different hypothesis including the implication of environmental factors, toxic algae and/or pathogens have been explored. In this context, oyster samples were collected from all affected locations in France and tested in order to identify pathogens. Different analyses were carried out including histology, bacteriology, PCR for the detection of OsHV-1 [26,27], *Vibrio splendidus *and *V. aestuarianus *[28], transmission electron microscopy (TEM), and experimental trials. Results of diagnostic tests indicated (Renault et al., unpublished data) that *(i) *there was no listed pathogen involved, *(ii) *OsHV-1 was detected in most of samples especially in moribund oysters, *(iii) V. splendidus, V. aestuarianus *and *V. harveyi *were also detected and *(iv) *virus particles looking like herpes viruses were observed by TEM in moribund tested oysters.

Experimental trials were also performed in order to reproduce mortality from naturally infected oysters collected in 2008 during mortality outbreaks and to test the hypothesis of infectious disease aetiology. In this context, an experimental protocol was developed. This protocol was based on *(i) *infectious tissue homogenates prepared from naturally infected oysters collected in the field during mortality outbreaks and *(ii) *intramuscular injection of these tissue homogenates in healthy appearing oyster spat. Mortality was daily monitored after injection and OsHV-1 DNA systematically researched by real time quantitative PCR. Some samples were also subjected to TEM analysis.

## Materials and methods

### Oysters

Three batches of moribund *Crassostrea gigas *oysters were collected on the field along the French coasts during abnormal mortality outbreaks in 2008 (Table 1). The first batch was composed of oysters collected at Gouville (Normandy - Cotentin), the second of oysters collected in the estuary of the Etel river (Brittany - Morbihan), and the last one corresponded to oysters collected on the site of Aulne located in the Bay of Brest (Brittany - Finistère). These animals were checked for OsHV-1 detection and served for the preparation of tissue homogenates. A control tissue homogenate was prepared from the batch of healthy appearing oysters used for experimental assays and showing no mortality in 2008 (see below; Table 1).

**Table 1:** Description of the four different initial tissue homogenates made from naturally OsHV-1 infected oyster spat collected on the field in France during the abnormal mortality outbreak in 2008

Name	Origin of animals	Virus DNA amount	OsHV-1 genotype
Homogenate 1	Normandy (Cotentin)	4.21 × 10^2^	μVar
Homogenate 2	South Brittany (Morbilhan)	2.18 × 10^3^	μVar
Homogenate 3	North Brittany (Bay of Brest)	2.03 × 10^2^	μVar
Homogenate 4	Mediterranean sea	0	-

Virus DNA amounts of oyster tissue homogenates were quantified by real-time quantitative PCR and expressed as the number of virus DNA copies per μL of water. OsHV-1 genotypes of homogenates were determined by sequencing of the virus genome.

Experimental infection trials were performed on healthy appearing *C. gigas *spat (one year old) purchased in November 2008 from a shellfish farm located on the French Mediterranean coast. No mortality event has been reported at this shellfish farm location during 2008. Oyster spat sized around 40 mm in length, with a mean weight of 5 grams. Oysters were placed in the Ifremer's facilities (Laboratoire de Génétique et Pathologie, La Tremblade, France) in a single tank of 200 L containing filtered (1 μm) seawater and slowly acclimated to 22°C increasing the temperature of 1°C per day. During this period, oysters were fed daily by addition of 2 liters of microalgae *Skeletonema costatum *(1.5 10^3 ^cells/mL). Oysters did not present any mortality or other symptom of disease at this time. At the end of the acclimatization period and just before the beginning of the experiment, a set of 20 individuals was assessed by real time quantitative PCR in order to evaluate the initial OsHV-1 DNA detection.

No ethical approval has been requested for the present study because experimental research has been conducted on Pacific oysters (invertebrates). Oysters don't possess a central nervous system.

### Preparation of oyster tissue homogenates

Initial tissue homogenates were prepared using ten animals from each batch (Table 1). Oysters were open by removing the superior valve. To verify that animals were OsHV-1 infected, a small piece of mantle (25 mg) was sampled from each individual used and frozen at -20°C before DNA extraction and OsHV-1 detection by real time quantitative PCR. Gills and mantle of these animals were then dissected and pooled together in a 50 mL sterile tube. All subsequent dilutions were made using 0.22 μm filtered artificial seawater (ASW). The total mass of tissues was weighted and 10 volumes of 0.22 μm ASW were added in the tube (9 mL of seawater per g of tissues). Tissues were then crushed on ice using an Ultraturax mixer (3 × 5 s). After centrifugation (1000 *g*, 5 min, 4°C), supernatant was placed in a new tube and diluted by addition of 4 volumes of ASW. Finally, the clarified tissue homogenate was filtered consecutively in sterile conditions using syringe filters at 5 μm, 2 μm, 0.45 μm and 0.22 μm pore sizes (Millipore; Billerica, USA).

Other filtered tissue homogenates were prepared using the same protocol from dying and dead animals after intramuscular injections. Similarly, control tissue homogenates were also prepared using healthy oysters (oysters from Mediterranean coast).

Filtered tissue homogenates were stocked at 4°C until use. A volume of 200 μL of each filtered tissue homogenate was sampled and stored at -20°C for further DNA extraction and OsHV-1 DNA quantification by real time quantitative PCR.

### Experimental design: intramuscular injection of filtered tissue homogenates

**Figure 1:**
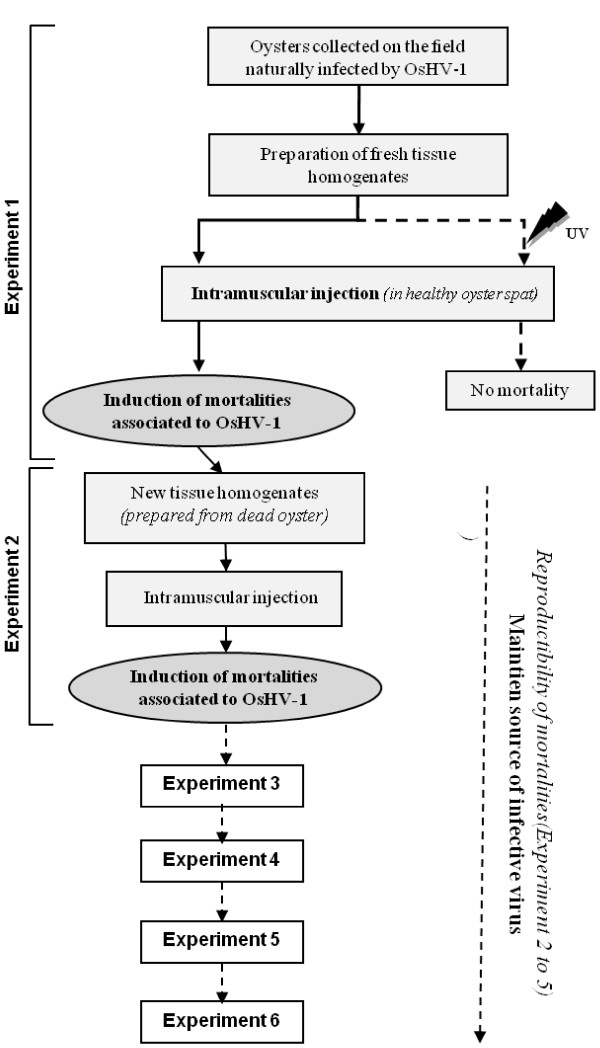
**Scheme of the experimental trials undertaken in the present study to reproduce virus OsHV-1 infection**. Experiments 1 and 2 were carried out using tissue homogenates prepared from naturally infected oysters. Experiments 3 to 6 were performed using tissue homogenates made from experimentally infected oysters.

Each experimental trial (6 assays were conducted in triplicates) was carried out using 8 healthy oyster spat (Experiment 1, Figure 1) or 10 healthy oyster spat (Experiment 2 to 6, Figure 1). Animals were first placed out of water for 24 h at 22°C and then anesthetized during 4 h in a solution containing 7% (w/v) magnesium chloride (MgCl_2_; 50 g/L) in seawater (1 v)/distilled water (4 v) [29]. Once a time relaxed, 100 μL of a filtered tissue homogenate exposed or not to an UV treatment were injected into the adductor muscle using a 1 mL micro-syringe equipped with 18-g needles.

Filtered tissue homogenates in Experiment 2 were subjected to a UV treatment adapted from Chan and Guilbert [30]. Samples were exposed 15 mm at 10 cm of a UV lamp (VL115-C, Vilber Lourmat, 15 W) emitting a dose of 1.08 mW/cm² at 254 nm.

Negative controls (Experiments 1 to 6) consisted of oysters intramuscularly injected with control tissue homogenates prepared from healthy spat. Inoculated oysters were then placed at 22°C in 5 L tanks supplied in filtered (1 μm) seawater without food supply. Mortality was daily monitored during a 7 day period. Dead oysters were removed from tanks during the time course of the experiments and a piece of mantle (25 mg) was sampled on each dead individual. Samples of mantle were also dissected on surviving oysters which were sacrificed at the end of experiments. All tissue samples were frozen at -20°C for further DNA extraction and OsHV-1 detection by real time quantitative PCR.

### DNA extraction

Total DNA was extracted from tissue fragments (mantle) and tissues homogenates (mantle + gills) using the QIAgen (Hilden, Germany) QIAamp tissue mini kit combined with the use of the QIAcube automate according to the manufacturer's protocol.

Briefly, samples of mantle were digested overnight on a rocking platform at 56°C by addition of 180 μL of ATL supplied buffer with 20 μL of proteinase K per 25 mg of sample. When the tissue was completely lyzed, total volume of 200 μL of lysate was transferred into a 2 mL microcentrifuge tube and DNA extraction with QIAamp Mini spin columns was carried out using a QIAcube automate. Final elution of DNA extracted from mantle samples was performed with 200 μL of double-distilled water.

For tissue homogenates, DNA was extracted from 200 μL of samples and performed manually with a final elution with 50 μL of double-distilled water.

DNA concentrations and DNA quality were measured using a spectrophotometer Eppendorf (Hamburg, Germany). Extracted DNA was stored at -20°C prior OsHV-1 detection and quantification by real time quantitative PCR.

### OsHV-1 DNA quantification by real time quantitative PCR

The detection and quantification of OsHV-1 DNA was carried out using real-time PCR [26]. After dilution at 2 ng/μL, 5 μL of DNA samples were added to the reaction mix composed of 12.5 μL of Brillant^® ^SYBR Green Master Mix reagent (Agilent; Santa Clara, USA), 2.5 μL of both C9 forward and C10 reverse primers [31] diluted at the concentration of 2 μm each, and 2.5 μL of distilled water. All amplification reactions were performed using a Mx3000P real-time PCR thermocycler (Agilent) with 96-microwell plates according to the following conditions: 1 cycle of pre-incubation at 95°C for 10 min; 40 cycles of amplification at 95°C for 30 s, 60°C for 1 min, and 72°C for 1 min; and a final step for melting temperature curve analysis at 95°C for 1 min, 60°C for 30 s, and 95°C for 30 s. The specificity of the PCR products was systematically checked with the melting temperature value calculated from the dissociation curve [32]. Absolute quantification of OsHV-1 DNA copies was carried out by comparing CT values obtained for tested samples with the standard curve based on a ten-fold dilution curve derived from a stock solution of OsHV-1 genomic DNA (5 × 10^6 ^copies/μL) extracted from purified virus particles [6]. Efficiency (E) and linearity (R²) were calculated from the standard curve with MxPro v3.0 software (Agilent), and tested for each run. All samples were analyzed in triplicate. The results were expressed as a log^10 ^of the virus DNA copy number per ng of total DNA or per μL of control or infectious homogenates.

### Transmission electron microscopy examination

Fragments of mantle, adductor muscle and digestive gland were collected from 3 moribund oysters after injection of filtered tissue homogenate (Experiment 1, Figure 1) and fixed in 2.5% (v/v) glutaraldehyde in 0.2 M cacodylate buffer (pH 7.2) for 2 h at 4°C. After 2 washes in 0.2 M cacodylate buffer (pH 7.2), samples were post-fixed in 1% (w/v) osmium tetroxide in the same buffer (1 h, 4°C). The samples were then washed in 0.2 M cacodylate buffer (pH 7.2) (2 × 15 min), dehydrated in a graded series of ethanol (70%, 10 min; 95%, 2 × 15 min; 100%, 3 × 20 min at room temperature), cleared in propylene oxide (2 × 20 min), infiltrated (propylene oxide/Epon resin (vol/vol), 1 h at room temperature; Epon resin, 1 h at room temperature) and embedded in Epon resin (48 h at 60°C). Ultrathin sections were cut using a Leica (Wetzlar, Germany) microtome, collected on copper grids and double stained with uranyl acetate and lead citrate using conventional techniques. Examination was carried out on a JEOL JEM 1011 (Tokyo, Japan) transmission electron microscope at 60 kV.

### Sequencing of OsHV-1 genome

OsHV-1 isolates were characterized by sequencing PCR products using primer sets targetting two regions of the virus genome, the region IA (ORF43) that encodes a putative apoptosis inhibitor and the region C (ORF4) that encodes two proteins of unknown function [33]. The first primer pair used IA1/IA2 (IA1: CGC GGT TCA TAT CCA AAG TT/IA2: AAT CCC CAT GTT TCT TG CTG) amplified a 607 bp fragment and the second primer pair, C2/C6 a (C2: CTC TTT ACC ATG AAG ATA CCC ACC/C6: GTG CAC GGC TTA CCA TTT TT) amplified a 709 bp fragment. The sequencing reaction was carried out into a 10 μL final volume, containing 1.8 μL of sequencing buffer, 0.4 μL of BigDye^® ^Terminator v3.1 (Applied Biosystems; Carlsbad, USA), 1.5 μL of primer Forward or Reverse at 4 μM, fresh purified PCR products and 10 μL sterile DNAse and RNAse free water. The program consisted of an initial denaturation of 3 min at 96°C followed by 35 cycles of 30 s at 96°C, 30 s at 50°C and 4 min at 60°C. This reaction was performed using a 96-well plate. Sequencing reactions were then purified as follows. In each sample, 60 μL of 100% ethanol were added and samples were centrifuged at 1500 *g *for 30 min. The plate was inverted to remove ethanol. Then, 60 μL 70% ethanol were added, followed by centrifugation of the plate at 1000 *g *for 10 min. The plate was then centrifuged upside down for 30 s to remove ethanol. Finally, samples were dried in a Speed Vac and re-suspended with 10 μL deionized formamide. Samples were loaded in ABI PRISM^® ^3130 XL-Avant Genetic Analyzer (Applied Biosystems), using a 36 cm capillary array and POP 7 polymer. Sequences were edited with Chromas lite 2.01 version software. A multiple alignment was achieved by Bioedit 7.0.9 version software using the algorithm CLUSTAL W. Sequence results were compared to the consensus sequence OsHV-1 available on GENBANK [accession number AY509253].

### Statistical analysis

Statistical analyses were carried out using the XLSTAT-Pro^® ^v7.5.3 software (Addinsoft; Paris, France). Mortality rates after injection of the different tissue homogenates were tested using a Khi² test. A non-parametric Kruskal-Wallis test followed by a Dunn post-hoc comparison test was performed to compare differences between means of virus DNA amounts (DNA copy number) quantified in dead or alive oyster tissues. A P-values level of 0.05 was used in all tests to identify signicant effects or differences.

## Results

### Inducing oyster mortality by tissue homogenate injection

In a first experiment (Experiment 1), four filtered tissue homogenates (Table 1) were tested by intramuscular injection in healthy oyster spat. Tissue homogenates 1, 2 and 3 were prepared using moribund oysters which proved to be naturally infected by OsHV-1 collected in Normandy (Cotentin), in South Brittany (Morbihan) and in the Bay of Brest (Finistère), respectively (Table 1). OsHV-1 DNA amounts quantified in filtered tissue homogenates 1, 2 and 3 were 4.21 × 10^2^, 2.18 × 10^3 ^and 2.03 × 10^2 ^DNA copies/μL, respectively. Moreover, sequence analysis revealed the presence of OsHV-1 μVar genotype in the three tissue homogenates [33]. No virus DNA was detected in the filtered tissue homogenate 4 which was prepared from a batch of healthy oysters collected from Mediterranean coast (Table 1).

**Figure 2:**
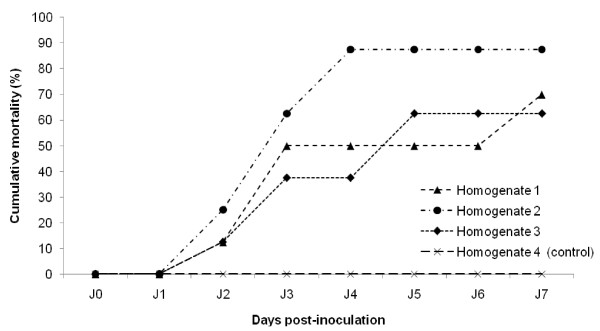
**Cumulative mortality of Pacific oyster spat recorded after injection of filtered (0.22 μm) tissue homogenates made from different batches of moribund oyster spat naturally infected by OsHV-1 and collected on the field**. Tissue homogenates 1, 2, 3 were made from moribund oyster spat collected in Normandy, South Brittany and the Bay of Brest, respectively. Homogenate 4 was made from healthy oyster spat from Mediterranean Sea. Results are represented as the percentage of cumulative mortality (*n *= 8).

For the first experiment (Experiment 1), mortality rates recorded in healthy oyster spat after intramuscular injection of tissue homogenates were represented on Figure 2. For tissue homogenate 1, the cumulative mortality rate reached 50% at day 3 post injection and remained stable until day 6, before to reach 70% at day 7. For tissue homogenate 3, the cumulative mortality rate reached 40% at day 3 post injection before to get around 65% at day 5 until the end of the experiment. Oyster spat intramuscularly injected with tissue homogenate 2 demonstrated a cumulative mortality rate of 25% 2 days post-injection. The mortality rate reached gradually 90% at day 4 and stayed stable until the end of the experiment. The mortality rates observed after injection of tissue homogenate 3 appeared significantly higher from the fourth day of the experiment to those observed with tissue homogenates 1 and 2. No mortality was observed in oyster spat injected with tissue homogenate 4, which constituted the negative control tissue homogenate (Figure 2).

**Figure 3:**
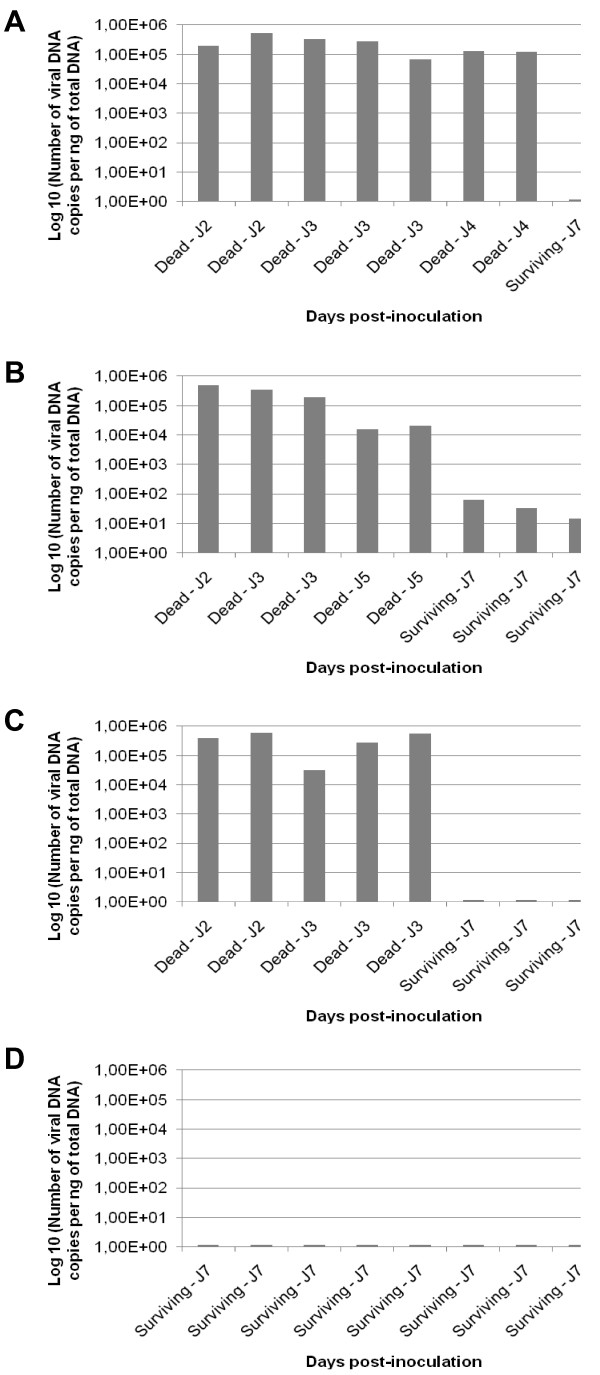
**Virus DNA detection by real time quantitative PCR in dead or surviving oyster spat after injection of the different filtered (0.22 μm) tissue homogenates made from moribund oyster spat naturally infected by OsHV-1 and collected on the field**. Tissue homogenates 1, 2 and 3 were made using moribund oyster spat collected in Normandy (**A**), South Brittany (**B**) and the bay of Brest (**C**), respectively. Tissue homogenate 4 was made using healthy oyster spat from Mediterranean Sea (**D**). Results are expressed as the mean number of virus DNA copies detected per ng of total DNA extracted from samples of mantle.

The quantification of OsHV-1 DNA was carried out in dying or dead individuals collected during the time course of the experiment (Experiment 1) and in surviving individuals collected at the end of the experiment (Figure 3). The average virus DNA amounts were 3.67 × 10^5^, 2.35 × 10^5 ^and 2.17 × 10^5 ^DNA copies/ng of total DNA extracted from mantle in dying or dead oysters injected with tissue homogenates 1, 2 and 3, respectively. Individual virus DNA amounts ranged from 3.19 × 10^4 ^to 5.93 × 10^5^, 6.67 × 10^4 ^to 5.23 × 10^5^, and 1.59 × 10^4 ^to 5.07 × 10^5 ^DNA copies/ng of total DNA extracted from mantle for dying or dead oysters, injected with tissue homogenates 1, 2 and 3, respectively. No virus DNA was detected in surviving individuals, except for oysters surviving after injection of the tissue homogenate 3 which presented a maximum virus DNA amount of 6.13 × 10^1 ^DNA copies/ng of total DNA extracted from mantle. No virus was detected in animals injected with the negative control tissue homogenate (tissue homogenate 4).

**Figure 4:**
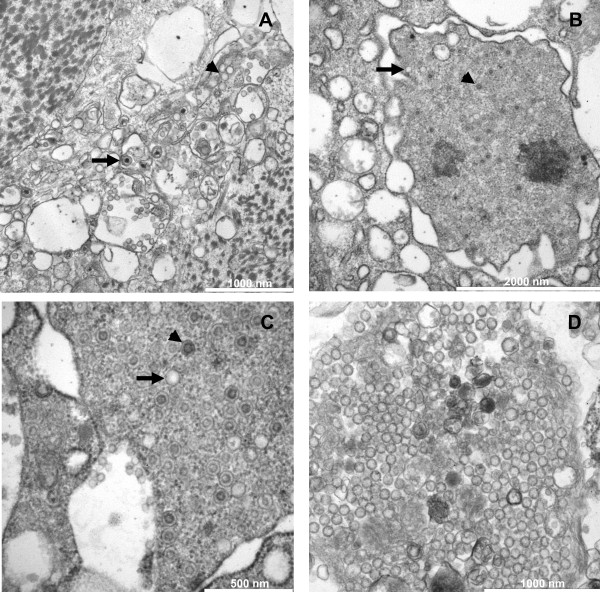
**Transmission electron micrographs of OsHV-1 infected tissues from Pacific oyster spat**. **A**: enveloped particles (arrows) and L-particles (arrows head) detected in adductor muscle tissue. Scale bar = 1 μm; **B**: capsids (arrows) and nuclocapsids (arrows head) detected in the nucleus of an infected cell in the mantle. Scale bar = 2 μm; **C**: high magnification of capsids (arrows) and nucleocapsids (arrows head) present in the nucleus of a mantle cell from an infected oyster. Scale bar = 500 nm; **D**: high magnification of L-particles detected in the mantle of an infected oyster. Scale bar = 1 μm.

Transmission electron microscopy of 3 moribund oysters after injection of tissue homogenate 1 (Experiment 1, Figure 1) revealed virus particles with morphology that closely resembled herpes viruses in the adductor muscle, the mantle (Figure 4,A and 4B) and the digestive gland. These oysters were collected two days after injection of the filtered (0.22 μm) tissue homogenate. Intra-nuclear empty capsids, nucleocapsids and enveloped particles were noticed (Figure 4,A and 4C). Moreover, numerous particles interpreted as Light particles (L-particles; capsidless particles) were also observed into perinuclear cisterna and cytoplasmic vesicles of infected cells (Figure 4,C and 4D). L-particles were spherical, ranged in size between 76 nm and 63 nm and contained homogeneous granular material of low electron density.

### Effect of UV treatment on oyster mortality induction

**Figure 5:**
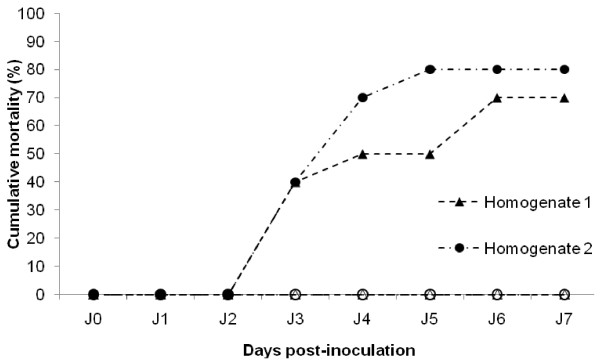
**Cumulative mortality of Pacific oyster spat recorded after injection of two different filtered (0.22 μm) tissue homogenates exposed or not to a UV treatment**. Tissue homogenates 1 and 2 were made from moribund oyster spat collected in Normandy and South Brittany, respectively. Results are represented as the percentage of cumulative mortality (*n *= 10).

The effect of UV treatment on the infectivity of tissue homogenates was investigated in a second experiment (Experiment 2). Injection of tissue homogenates 1 and 2 induced cumulative mortality rates of 40% three days post-injection before to reach 70% and 80% at the end of the experiment on day 7, respectively (Figure 5). No mortality was recorded among oysters injected with tissue homogenates 1 and 2 previously exposed to an UV treatment (Figure 5).

**Figure 6:**
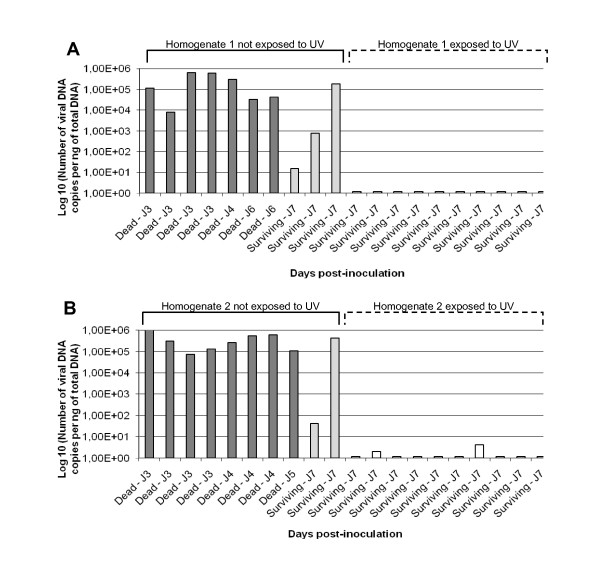
**Virus DNA detection by real time quantitative PCR in dead or surviving oyster spat after injection of two different filtered (0.22 μm) tissue homogenates exposed or not to a UV-treatment**. Tissue homogenates 1 and 2 were made with moribund oyster spat collected in Normandy (**A**) and South Brittany (**B**), respectively. Results are expressed as the mean number of virus DNA copies detected per ng of total DNA extracted from samples of mantle.

The virus DNA quantification was determined in dying or dead individuals collected during the time course of the experiment (Experiment 2), (Figure 6). The average virus DNA amounts were 2.51 × 10^5 ^and 3.91 × 10^5 ^DNA copies/ng of total DNA after injection of tissue homogenates 1 and 2, respectively. Individual OsHV-1 DNA amounts ranged from 7.83 × 10^3 ^to 6.55 × 10^5 ^and 1.51 × 10^1 ^to 1.86 × 10^5 ^DNA copies/ng of total DNA extracted from mantle after injection of tissue homogenates 1 and 2, respectively.

Virus DNA was also detected in surviving individuals collected at the end of the experiment with values reaching up to 1.86 × 10^5 ^and 4.19 × 10^5 ^DNA copies/ng of total DNA after injection of tissue homogenates 1 or 2, respectively (Figure 6). Statistical analysis revealed that the average virus DNA amounts detected in the mantle of oyster spat were not significantly different in dying or dead individuals after injection of tissue homogenates 1 and 2. No virus DNA was detected in oyster spat surviving to the injection of tissue homogenate 1 previously exposed to UV. Only two individuals were positive for OsHV-1 DNA detection in individuals injected with tissue homogenate 2 previously exposed to UV at very low levels (1.97 and 4.28 OsHV-1 DNA copies/ng of total DNA).

### Reproducing mortalities in experimental conditions

**Figure 7:**
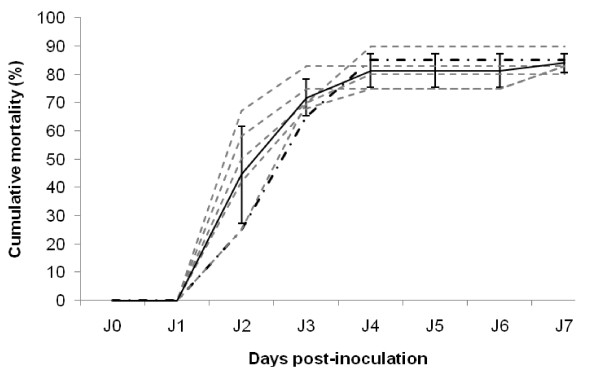
**Reproducibility of mortality on Pacific oyster spat after injection of different infectious OsHV-1 homogenates made from experimentally infected animals**. The dash line represent the mortality recorded after injection of the initial homogenate 2, which is made from naturally infected oyster spat collected on the field (South Brittany). The dotted lines corresponds to mortalities recorded in five consecutive transmission assays conducted using five different homogenates made from oyster spat dead previously in experimental conditions. The mean mortality is indicated by the solid line. Results are expressed as the percentage of cumulative mortality (*n *= 10). Standard deviation is represented by error bars.

To demonstrate the reproducibility of inducing oyster mortalities in experimental conditions by intramuscular injection of tissue homogenates containing OsHV-1 DNA, four successive experimental transmission trials (Experiments 3 to 6) were carried out (Figure 1). Each experiment was carried out by injecting in the adductor muscle of healthy oyster spat a tissue homogenate prepared from dead oysters collected during the previous experimental assay. Dying or dead oysters collected after intramuscular injection of the initial tissue homogenate 2 were used to prepare the first tissue homogenate from experimentally infected oysters (Figure 1). The mean mortalities recorded during the four consecutive experimental transmission trials were around 40% two days after injection (Figure 7). Mean mortality rates increased gradually to reach 80% at day 4 and 85% at the end of the experiments at day 7. No statistical differences were evidenced between mean mortality rates recorded during these four consecutive experimental transmission trials and the mortality rates previously reported after the first intramuscular injection of the tissue homogenate 2 (Experiments 1 and 2). Moreover, the mortality rates reported at each monitoring point were not significantly different between the four consecutive trials.

**Figure 8:**
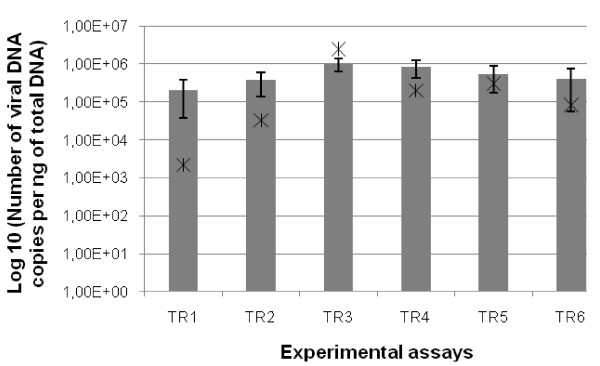
**Average virus DNA amounts estimated in the mantle of oyster spat dead in five consecutive transmission assays conducted using five different homogenates made from oyster spat dead previously in experimental conditions**. Stars represent virus DNA amounts of the different homogenates used in these consecutive transmission assays. Virus DNA amounts were quantified by real-time quantitative PCR. Standard deviation is represented by error bars.

Average virus DNA amounts quantified in the mantle of dying or dead individuals collected during experimental transmission assays were estimated at 1 × 10^6^, 8.32 × 10^5^, 5.37 × 10^5 ^and 4.01 × 10^5 ^DNA copies/ng total DNA, respectively (Figure 8). These virus DNA amounts were not significantly different between the different trials. The quantification of OsHV-1 DNA was also carried out in each different tissue homogenates used. Virus DNA amounts of 2.50 × 10^6^, 2.01 × 10^5^, 3.02 × 10^5 ^and 8.25 × 10^4 ^DNA copies/μL were detected in tissue homogenates injected during the four experimental transmission assays, respectively.

## Discussion

Significant cumulative mortality rates (around 80%) were observed in healthy oyster spat intramuscularly injected with filtered (0.22 μm) tissue homogenates prepared from naturally OsHV-1 infected oysters (Experiments 1 and 2). First mortality was reported two days after injection. As suggested by Sauvage et al., this period may correspond to an intense replication phase leading to irreversible cell damages resulting in mortality [34].

In addition, tissue homogenates prepared from experimentally infected oyster spat induced also high mortality rates (Experiments 3 to 6) demonstrating the reproducibility of the experimental protocol. Finally, filtered tissue homogenates exposed to UV did not induce any mortality. These results suggest that induced mortality in experimental conditions was related to the presence in oyster tissue homogenates of an infectious agent under 200 nm in size and sensitive to UV treatment. Filtration at 0.22 μm of tissue homogenates is used in order to retain bacteria that might be present in tissues of moribund oysters. OsHV-1 appears as an ideal candidate due to its size and its envelop. As OsHV-1 is an enveloped virus, it may be assumed that it is fragile. High temperature, chemicals and UV [35,36] may destroy its lipid containing envelope. The virus envelope is needed for herpes virus entry in target cells. However, tissue homogenates might also contain other viruses or substances such as bacterial toxins able to induce mortality [37].

Real-time quantitative PCR analysis of intramuscularly injected oysters revealed the presence of high amounts of OsHV-1 DNA suggesting that active virus replication occurred in injected oyster. Moreover, TEM examination demonstrated the presence of herpes virus particles in mantle, adductor muscle and digestive gland of moribund oysters and confirmed real time quantitative PCR results. Virus particles demonstrated all the characteristics of OsHV-1 [11,16,38]. Moreover, numerous particles interpreted as L-particles were also detected in moribund oysters. Although L-particles were not previously reported in the existing body of literature on mollusc herpes viruses, such particles have been already described for vertebrate herpes viruses including herpes simplex virus 1 and 2, and pseudorabies virus [39-41]. L-particles are capsidless particles and lack the virus genome, but appear to contain all virus envelop and tegument proteins. Finally, in examined samples no virus particles morphologically related to virus families other than herpes viruses were observed by TEM.

Four postulates for the establishment a causal relationship between a pathogen and a disease have been first proposed by Koch in 1890 [42]. These postulates require that *(i) *the microorganism must be present in abundance in all organisms suffering of the disease, but absent from healthy organisms; *(ii) *the microorganism must be isolated from moribund organisms and cultivated in vitro; *(iii) *the cultivated microorganism should induce the development of the disease when introduced into a healthy organism; *(iv) *the organism must be isolated again from the new host organism infected and identified as being identical to the original infectious agent. Our results full filled at least three of the four Henlé-Koch postulates [43]. The virus was detected in large amounts in oysters collected in the field during mortality outbreaks (1st postulate). Although the virus was not cultivable in vitro at present due to the lack of susceptible cell lines, the injection of filtered (0.22 μm) tissue homogenates prepared using naturally OsHV-1 infected spat induced high mortality rates in healthy oysters (3rd postulate). Finally, OsHV-1 was again detected based on real time quantitative PCR and TEM in injected oyster spat (4th postulate). Real time quantitative PCR analyses [26] showed that high amounts of virus DNA (approximately 1 × 10^5 ^DNA copies/ng of total DNA) were detected in the mantle of dying/dead oysters after intramuscular injection. As previously reported [16,26,34,44], the mantle appears as a target organ for OsHV-1 replication and may be recommended for epidemiological studies. Moreover, virus DNA amounts comparable to those quantified in the mantle of dying/dead individuals were detected in some surviving oysters at the end of the experiments. Such a result confirmed that all animals were infected after injection of tissue homogenates. In our study, an intramuscular route of infection was chosen to ensure that all experimental oysters were contaminated by the virus at the same time. Surviving oysters at the end of experiments (day 7 post-injection) may represent individuals collected before they died and/or be considered as less susceptible animals which developed the virus infection less rapidly. These results suggest that the susceptibility to the disease varies among oysters related to genetic diversity.

In this context, OsHV-1 may interpreted as the causative agent of oyster spat mortality noticed after filtered (0.22 μm) tissue homogenate injections. In addition to transmitting OsHV-1 infection by intramuscular inoculation, the virus can be also waterborne transmitted to healthy oyster spat, via co-habitation of healthy oyster spat with experimentally infected individuals [45].

Previous experimental works have been able to strengthen a causal link particularly concerning *C. gigas *larval stages. It was established that OsHV-1 induced larval mortalities [24,46,47]. To date, attempts to reproduce the virus infection in oyster spat have been inconclusive [25]. A first experimental data set indicated that OsHV-1 could be transmitted to healthy *C. gigas *spat through cohabitation with experimentally infected larvae when spat were kept in stressful conditions (Renault, unpublished data).

The intra-muscular injection of tissue homogenates prepared from infected oysters was used successfully in the present study. The capacity to reproduce the virus disease in the present study may be due to *(i) *the route of contamination *(ii) *the use of fresh infected oysters as the virus source and/or *(iii) *the use of a particular virus genotype (OsHV-1 μVar) presenting a high virulence. Although two ostreid herpesvirus 1 genotypes were previously characterized in France (OsHV-1 reference genotype and OsHV-1 var genotype) [11], the presence of a third genotype, termed OsHV-1 μVar, was reported in France in 2008 and 2009 in association with abnormal mortality outbreaks among French *C. gigas *[33]. Both OsHV-1 genotypes (reference and μVar) were detected in association with mortality outbreaks in 2008. Otherwise, the data collected in 2009 during French oyster mortality outbreaks demonstrated only the genotype OsHV-1 μVar detection. These results raise questions about the emergence and the virulence of OsHV-1 μVar genotype. Moreover, L-particles were detected in moribund oysters and may be related to increased virulence. Although the exact role of herpes virus L-particles remains to be determined, some authors suggested that these particles supply functions which can boost successful initiation of the infectious process under adverse conditions increasing infectivity and may have a role in vivo in enhancing reactivation from latency [39,48]. However, some experimental trials performed in 2008 with the reference like genotype, based on two ORF sequences characterization, showed that mortality could also be induced (data not shown). Further work is needed to fully investigate possible infectivity and virulence differences between OsHV-1 genotypes.

However, the demonstration of a causal link between a pathogen and mortality outbreaks in the field is not only based on the possibility of reproducing the disease experimentally, but should take into account the idea that the cause of mortality outbreaks can also be a combination of factors. A causal link between OsHV-1 infections and oyster spat mortalities has been suggested in different epidemiological studies on summer oyster mortalities worldwide [25,49,50]. In France, this causal link has been highlighted through an epidemiological monitoring over several years conducted by the National Network for Surveillance and Monitoring of Mollusc Health (unpublished data), which is based on a passive surveillance and laid down on oyster mortality declarations by shellfish farmers.

Although the absence of bivalve cell lines does not allow to cultivate the virus in vitro [25], the protocol developed in the present study allows to produce tissue homogenates containing infective virus particles. Preliminary assays have showed that tissue homogenates could be stocked at 4°C for one month keeping infectivity. During this time of storage, real time quantitative PCR analyses showed that the virus DNA amounts did not vary (data not shown). However, real time quantitative PCR does not allow the quantification of infective virus particles, which are necessary to initiate the virus infection in host-cells [51]. Nevertheless, our protocol may allow to maintain sources of infective virus particles through repeated experimental infections of healthy oyster spat. However, care must be taken that the virus does not loss its infectivity through successive in vivo passages.

The present study described an experimental protocol allowing to prepare tissue homogenates containing infective OsHV-1 particles. Results showed that it is possible to maintain sources of infective virus and contributed to the set up of a reproducible model for studying the infectivity of OsHV-1 in *C. gigas *oysters in experimental conditions, which can be useful to obtain information including potential mechanisms of transmission, entry routes, virus stability and virus-host interactions.

## Competing interests

This work was exclusively supported by Ifremer (Institut Français pour l'Exploitation de la Mer). The authors declare that they have no competing interests.

## Authors' contributions

This study is the result of a collective work. All authors conceived this study, and participated in its design. DSa, JFP and TR carried out the development of the protocol for the preparation of infectious tissue homogenates, with the help of others authors. DSc, NF, PM, TR and JFP carried out experimental pathology assays and molecular analysis. TR carried out the TEM examination. DSc performed the statistical analysis and drafted the manuscript with the help of TR. All authors read, corrected, and approved the final manuscript.
